# Exploring Potential Biomarkers in Recurrent Pregnancy Loss: A Literature Review of Omics Studies to Molecular Mechanisms

**DOI:** 10.3390/ijms26052263

**Published:** 2025-03-04

**Authors:** Lan Li, Kwang-Hyun Baek

**Affiliations:** 1 The Key Laboratory for Preclinical and Basic Research on Chronic Diseases, School of Basic Medical Sciences, North China University of Science and Technology, Tangshan 063210, China; dreamerlanlan@gmail.com; 2 Department of Biomedical Science, CHA University, CHA General Hospital, Seongnam-si 13488, Gyeonggi-do, Republic of Korea

**Keywords:** multi-omics, inflammation, infertility, signaling pathway, recurrent miscarriage

## Abstract

Recurrent pregnancy loss (RPL) is characterized by the occurrence of three or more consecutive spontaneous pregnancy losses before 20–24 weeks of gestation. Despite significant progress in the investigation of the biological pathways associated with unexplained RPL, the precise molecular mechanisms remain elusive. Recent advances in multi-omics approaches have identified numerous biomarkers that offer potential avenues for understanding the underlying complexities of RPL. The aim of this comprehensive literature review was to investigate the functional roles of these candidate markers and explore the possible key mechanisms that may contribute to RPL. We also aimed to elucidate the functional networks predicted by omics analyses, which hold promise for providing invaluable insights into novel diagnostic and therapeutic strategies for women experiencing RPL. Furthermore, this review expands on clinical implications and possible applications, highlighting those currently moving towards clinical use and ongoing studies developing in this direction.

## 1. Introduction

**Figure 1:**
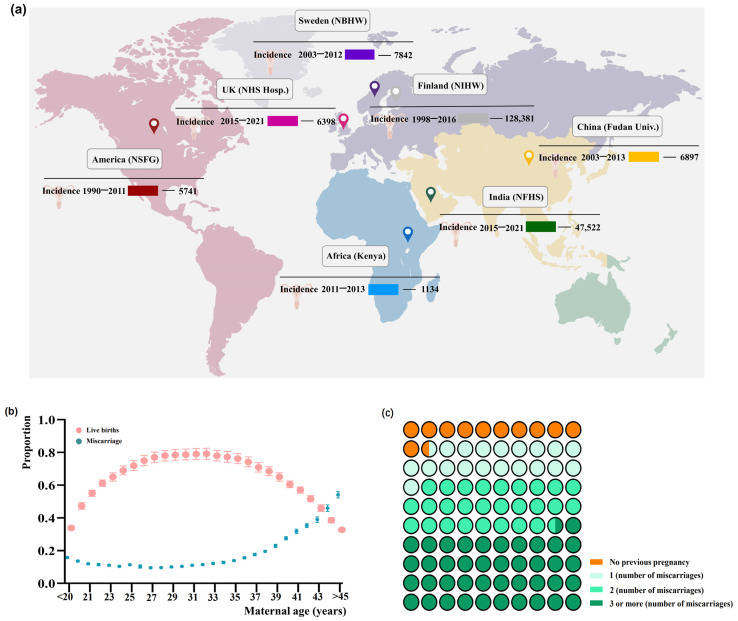
Global trends of pregnancy loss incidence. (**a**) A global map illustrating the incidence of pregnancy loss worldwide. (**b**) Population-based study depicting the frequency of pregnancy outcomes by maternal age, showcasing a J-shaped curve (green) indicating the age-associated risk of miscarriage and a parabola graph (the light pink curve) displaying the relationship between age and live births. The data exhibit odds ratios with 95% confidence intervals (bars). (**c**) Segment of the comprehensive chart revealing the recurrence risk of pregnancy loss after consecutive miscarriages, listed in order from “no” to “more”: no pregnancy loss, one miscarriage, two consecutive miscarriages, and three or more consecutive miscarriages. “No miscarriage” makes up approximately 11.6% of all pregnancies, while “three or more consecutive miscarriages” accounts for about 41.9%. Reprinted from [10].

Recurrent pregnancy loss (RPL) is typically defined as two or more clinically recognized miscarriages before the 24th week of pregnancy. However, the European Society of Human Reproduction and Embryology (ESHRE) recently revised its definition to include two or more consecutive pregnancy losses, though this criterion varies across different countries (Figure 1a). Large-scale studies conducted in Europe and the USA suggest a correlation between advancing maternal age and an increased risk of recurrent miscarriage (Figure 1b). Yet, the risk of miscarriage increases with each successive pregnancy loss (Figure 1c) [1,2,3,4,5,6,7,8]. Known causes of RPL include karyotypic abnormalities, uterine malformations, thrombophilia, metabolic factors, endocrinologic disorders, and autoimmune disorders [9,10]. However, approximately 50–70% of RPL cases remain unexplained, leaving the pathophysiology largely unknown. Comprehensive evaluation of the causes of repeated miscarriages is controversial, with recommendations for thorough understanding of each pregnancy loss to determine appropriate patient evaluation and standardization. Alternative approaches targeting various molecules are actively being pursued for investigating and managing recurrent miscarriages.

**Figure 2:**
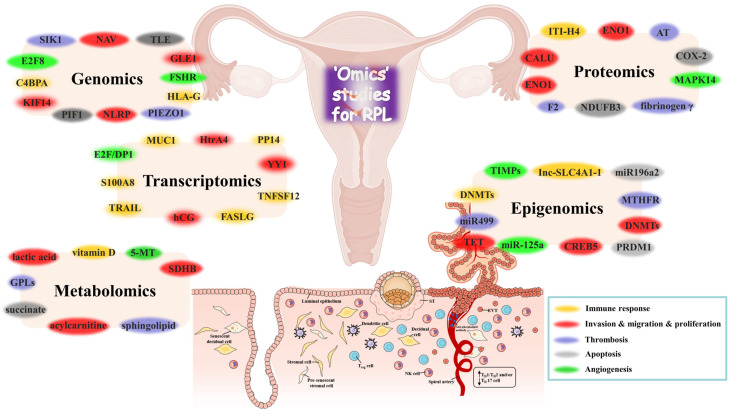
Schematic illustration of omics techniques in RPL. Different colored ovals represent types of functional omics annotations. Candidate targets related to RPL are involved in multiple pathways, including immune response, cell invasion/migration/proliferation, thrombosis, apoptosis, angiogenesis, and others. However, many of these targets remain to be identified for their detail functions with respect to the pathophysiology of RPL. Abbreviations: TRAIL, TNF-related apoptosis-inducing ligand; DNMTs, DNA methyltransferases; PP14, placental protein 14; TETs, ten-eleven translocation enzymes; TNFSF12, TNF superfamily member 12; MUC1, mucin 1; hCG, human gonadotrophin; ITI-H4, inter-α-trypsin inhibitor heavy chain family member 4; GPLs, glycerophospholipids; SIK1, salt-inducible kinase; F2, prothrombin; AT, antithrombin; NAV, neuron navigator; ENO1, enolase 1; CALU, calumenin; PRDM1, positive regulatory domain containing 1; CREB5, cAMP responsive element binding protein 5; YY1, Yin Yang 1; SDHB, succinate dehydrogenase complex iron sulfur subunit B; NDUFB3, NADH: Ubiquinone oxidoreductase subunit B3; TLE, transducer-like enhancer of split; 5-MT, 5-methoxytryptamine; MMPs, matrix metalloproteinases; E2F8, E2F transcription factor 8; MAPK14, mitogen-activated protein kinase 14; TIMPs, matrix metalloproteinases inhibitors of matrix metalloproteinases; GLE1, gametic lytic enzyme 1; FSHR, follicle stimulating hormone receptor; HLA-G, human leukocyte antigen G; PIEZO1, piezo type mechanosensitive ion channel component 1; NLRP, nucleotide-binding oligomerization domain, leucine rich repeat and pyrin domain containing; KIF14, kinesin family member 14; C4BPA, complement component 4 binding protein α.

Emerging qualitative methodological approaches, coupled with detailed explorations of affected families’ clinical histories, provide novel insights into the molecular pathogenesis of RPL. Recent omics data have identified new potential biomarkers characterizing key molecules and enhancing our understanding of the molecular mechanisms of RPL (Figure 2) [11,12]. Next-generation sequencing has broadened our ability to identify genes linked to RPL, particularly those related to cell division, ciliary function, and fetal movement. Comparative transcriptomic and epigenetic analyses of trophoblast–decidual tissue have revealed associations with DNA methylation and long-noncoding RNAs (lncRNA)-related immunoendocrine pathways in RPL [13,14,15,16]. Functional proteomic analysis has unveiled novel cellular mechanisms involved in RPL [17]. Integrating multi-omics approaches and functional insights promises to identify specific risks contributing to RPL in affected couples. This review discusses omics components underlying RPL’s molecular mechanisms, highlighting the advent of multi-omics in discovering RPL biomarkers and their clinical applications and implications.

## 2. Omics-Based Analysis of Recurrent Miscarriage

### 2.1. Genetics and Epigenetics in the Characterization of RPL

Genetic aberrations play a crucial role in the development and progression of recurrent miscarriages by disrupting essential biological pathways [18]. Accumulating data suggest that genetic variants and epigenetic modifications may be risk factors for miscarriage. These include copy number variations, DNA methylation patterns, abnormal microRNA expression, histone modifications, and noncoding regulatory RNAs (Table 1) [19,20]. This section addresses the correlation between RPL and genetic mechanisms, shedding light on potential therapeutic treatments for this complex reproductive challenge.

#### 2.1.1. Genetic Association Studies of RPL

The primary focus of genetic studies on recurrent miscarriages has been the discovery of DNA variants that may predispose individuals to abortion. Thus far, extensive research has examined numerous genes and single nucleotide polymorphisms (SNPs) [53,54]. Case-control studies, which commonly target genes associated with immune tolerance, blood coagulation, and maternal metabolism, have been the predominant approach used to investigate recurrent miscarriage. However, owing to inconsistent outcomes and limited clinical values, recent guidelines have dropped the recommendation to test the common SNPs associated with RPL risk. Genome-wide association studies (GWASs) have identified multiple loci associated with RPL, involved in ovarian progesterone production, placenta formation, and gonadotropin regulation [18,21,22,23,55]. Whole-exome sequencing has revealed 14 candidate variants, representing 43% of those with primary infertility and 13% of those with RPL. These variants include *TLE6*, *NLRP7*, *FSHR*, *ZP1*, *NLRP5*, *DNAH11*, *CCNO*, *CCDC68*, *CBX3*, *CENPH*, *PABPC1L*, *PIF1*, *PLK1*, and *REXO4* [22]. In addition to GWASs and replication studies, family-based association studies and subsequent candidate gene investigations have been conducted across different ethnic populations to examine common SNPs that could influence protein expression or function [24,25]. Chromosomal microarray analysis, either array comparative genomic hybridization or SNP-array-based, is the preferred method for profiling genome-wide chromosomal aberrations in patients with RPL owing to its high resolution and analytical standardization in the detection of structural variants. Additional data on multiple rare maternal–fetal variants linked to RPL in the era of exome sequencing and genome sequencing are expected to emerge over the next 5–10 years. The identification of pathogenic variant-enriched regions among patients with RPL and the discovery of novel etiologies are expected to accelerate the adoption of exome sequencing as a molecular diagnostic tool in the routine management of couples with RPL.

#### 2.1.2. Gross Epigenetic Disturbances of RPL

1. Methylation

Substantial epigenetic alterations, especially in unexplained recurrent pregnancy loss (URPL), may represent critical changes in epigenetic markers during pregnancy, potentially contributing to RPL pathogenesis. Therefore, studies on the role of aberrant epigenomic programming in recurrent miscarriage are necessary. Nevertheless, numerous technical and clinical challenges persist in the collecting suitable biomaterial samples for the epigenetic profiling of early pregnancy loss in humans. Published reports on human placental samples and retained products of conception epigenomes are limited. The most studied epigenetic mechanisms involve DNA methylation in promoters and genome-wide expression profiles interpretation. Abnormal DNA methylation and related enzymes, including DNA methyltransferases (DNMTs) and ten-eleven translocation enzymes (TETs), are associated with abnormal immune tolerance, failed cell invasion, and abnormal placental development, potentially contributing to URPL [26,27,56]. Abnormal methylation of the *5,10-methykenetetrahydrofolate reductase (MTHFR)* gene, which is involved in folate metabolism and thrombophilia, may affect embryo development and potentially lead to early miscarriage. However, there is ongoing controversy over whether folic acid supplementation can mask or effectively treat methylation defects in MTHFR-related RPL patients [1]. Analysis of human chorionic villi and decidual tissues from patients with RPL has shown downregulation of DNMT expression levels involved in DNA methylation, along with upregulation of DNA demethylase TETs [57]. Consistent with the findings on impaired epigenetic reprogramming, the decidual/endometrial tissue of recurrent miscarriage also exhibit lower expression levels of principal epigenetic “writer” enzymes, including methylated histone (H3-K9) and methyl transferase (G9aMT) [28]. In the context of imprinted genes associated with RPL, studies have implicated *DLK1*, *PEG10*, *PLAGL1*, *KCNQ1OT1*, *PEG3*, *GRB10*, and *PEG1/MEST* in women experiencing spontaneous abortion [58,59]. Abnormal DNA methylation resulting from defects in imprinted genes, including *GRB10* and *IGF2*, may contribute to fetal metabolic, neurological, and embryonic developmental disorders [29,30,60].

Human gonadotrophin (hCG), also known as chorionic gonadotropin-β5 or chorionic gonadotropin-β8, exhibits hemi-methylation, and its secretion is decreased in women with RPL. In addition, hypomethylation of PRDM1 is associated with the pathogenesis and progression of RPL [61]. PRDM1 differentially methylated regions regulate trophoblast cell apoptosis and migration by recruiting transcription factors such as GATA2 and FOXA1. In normal pregnancies, extensive angiogenesis occurs at the maternal–fetal interface to supply oxygen and nutrients to the embryo [62]. Consequently, abnormal angiogenesis is closely linked to pregnancy complications, such as early pregnancy loss and fetal growth restriction [63]. Histone trimethylation at the promoter regions of matrix metalloproteinases (MMPs) and their inhibitors are involved in RPL through various epigenetic mechanisms [64]. Recent studies have integrated genome-wide DNA methylation with RNA sequencing analysis of endometrial tissues and proposed novel epigenetic pathways. Hypomethylation of cAMP-responsive element binding protein 5 increases its expression by recruiting other transcription factors, causing the dysregulation of trophoblast cells, leading to recurrent miscarriages [31].

2. MicroRNAs

In recent years, research efforts related to RNAs, including microRNAs (miRNAs) and lncRNAs, and their implications in RPL have increased [32,33,65,66]. Currently, approximately 1500 miRNAs have been identified, targeting more than one-third of human genes involved in the regulation of normal pregnancy. MiR-125a, associated with RPL, regulates the expression of leukemia inhibitory factor receptor, contributing to embryo–endometrium crosstalk [67]. Combinations of mutations in miR-25, miR-32, miR-125, and miR-222, which regulate TGF-β signaling, are associated with an increased risk of RPL [32]. In addition to TGF-β, these genes are likely to interact with genes associated with angiogenesis, vasculogenesis, and thrombosis (VEGF and fibrinogen), further contributing to RPL pathogenesis. Structural variations and epigenetic signatures likely contribute to the genetic background of RPL. Individual miRNAs regulate multiple downstream target genes, complicating their spectrum of effects. MiR-146a attenuates apoptosis and regulates *FAS* mRNA expression [68], while NF-κB upregulates miR-146a, promoting the survival of mesenchymal stem cells. However, the specific effects of miRNA gene mutations on the development of RPL remain unclear. Therefore, using combinations of relevant miRNA genes for assessment may aid in identifying biomarkers for diagnosing RPL more effectively than evaluating a single gene.

3. Long-noncoding RNAs

lncRNAs represent additional special factors in the epigenetic control of target gene expression, and studies on these RNAs may contribute to understanding the mechanisms involved in recurrent miscarriage [35]. Recent studies have identified the differential expression of lncRNAs in the fetal sac and decidua in recurrent miscarriage and induced abortion [35]. The dysregulation of imprinted IncRNAs controlled by DNA methylation primarily involves pathways related to endocrine function, cellular–extracellular matrix interactions, immune responses, and apoptosis, all of which may be implicated in RPL. lnc-SLC4A1-1 is highly expressed in chorionic villi derived from patients with URPL [34]. lnc-SLC4A1-1 can bind to NF-κB, enhancing the expression level of CXCL8, which subsequently induces IL-1β and TNF-α secretion, thereby exacerbating trophoblast inflammation [34]. Although basic research on RPL epigenetics has provided substantial novel insights, the integration of epigenetic testing into clinical treatment and counseling for RPL cases is yet to be established.

### 2.2. Transcriptomic Factors Associated with RPL

Over the past decade, numerous studies have investigated the transcriptome of chorionic villi in an effort to identify novel biomarkers with predictive potential for recurrent miscarriage. Transcriptomics analyses, using oligonucleotide microarrays and high-throughput RNA sequencing (RNA-Seq), have become essential tools for profiling RNA expression patterns in RPL (Table 1) [69,70]. Various microarray platforms have been employed to investigate gene expression associated with RPL in experimental models. A PCR-based subtractive hybridization analysis showed downregulation of immune-related genes, including *human gonadotrophin (hCG)*, *placental protein 14 (PP14)*, and *mucin 1 (MUC1)*, in chorionic villi from patients with RPL compared to normal controls [71]. *High-temperature requirement factor A4 (HtrA4)*, a promising candidate gene, is significantly downregulated in patients with RPL during early gestation [14]. Reduced HtrA4 expression has been implicated in abnormal trophoblast physiological events that potentially affect the placental functions [36]. The patterns of RNA expression in RPL have been studied to identify biomarkers for disease progression and obtain insights into RPL pathogenesis. Various sample types, including decidua, villi, and blood from therapeutic abortion and recurrent spontaneous abortion cases, have been analyzed. Chorionic villus sampling remains the primary method for transcriptomic analysis of RPL. The transcriptomic profiles of chorionic villi from patients with RPL reflect malfunctions in basic nuclear processes and potential organismal feedback mechanisms to rescue the fetus. In a microarray study, increased expression levels of the TNF-related apoptosis-inducing ligand (TRAIL) and S100A8, encoding the inflammatory marker calprotectin, were observed in placental tissues from recurrent miscarriage cases, reflecting fetal rejection and abnormalities at the maternal–fetal interface during early pregnancy [37,38]. Compared with microarrays, RNA-Seq provides a higher resolution for profiling the entire transcriptome [38]. A recent RNA-Seq dataset of samples from normal and RPL pregnancies revealed that the majority of the differentially expressed genes in the chorionic villi of patients with RPL could be regulated via the transcription factor E2F and its functional partner DP1. The analysis linked the E2F/DP1 pathways to endoreplication, placental development, and fetal viability [39]. Transcriptomic analyses at the single-cell level using decidual samples from patients with RPL and healthy controls revealed that the upregulation of TNF superfamily member 12 (TNFSF12) and FASLG in RPL might be related to an abnormal decidual stromal niche and contribute to pregnancy failure [40]. A systematic analysis of aberrations in recurrent miscarriage transcriptomes and proteomes can illuminate the biological mechanisms involved in the initiation or promotion of miscarriage. However, while the transcriptomics of RPL have been extensively discussed, limited studies have investigated transcriptome-wide gene expression signatures using biomaterials collected from patients with RPL.

### 2.3. Metabolomics Studies in RPL

Pregnancy entails physiological changes and metabolic adaptations crucial for fetal development. Thus, there has been an increased focus on metabolomic research to address pregnancy-related concerns. Metabolic disorders during pregnancy, such as preeclampsia and recurrent pregnancy loss (RPL), can have potentially adverse long-term consequences [72]. Moreover, endocrinological abnormalities, including thyroid dysfunction, polycystic ovarian syndrome (PCOS), prolactin deficiency, luteal phase insufficiency, disturbances in insulin resistance, vitamin D deficiency, hyperandrogenism, and hyperhomocysteinemia, may also contribute to recurrent miscarriages [73,74,75]. Overt hypothyroidism is a well-established risk factor for miscarriage and fetal brain development [76,77]. The debate persists as to whether women with consecutive RPL have a higher prevalence of subclinical hypothyroidism compared to the prevalence of those without RPL [78].

Furthermore, PCOS is associated with RPL and other pregnancy complications, including gestational diabetes, pregnancy-induced hypertension, and preeclamptic toxemia [1]. Although PCOS may not be an independent risk factor for pregnancy loss, the miscarriage rate appears to be influenced by PCOS-related comorbidities [79,80]. Recent observational studies have suggested that obese women with a history of recurrent miscarriages face a high risk of future pregnancy loss, a risk not identified in overweight women [81]. Within a cohort of women with PCOS, a clear association was observed between the level of weight loss and the rate of miscarriage [82]. Prolactin deficiencies, possibly related to obesity, luteal phase insufficiency, PCOS, or stress, complicate studies that attempt to establish a direct connection between prolactin and recurrent miscarriages [83,84].

Recent untargeted metabolomic profiling has revealed significant alterations in pregnancy-related metabolites and metabolic pathways during healthy pregnancies. Noteworthy changes include variations in steroid hormone biosynthesis and arachidonic acid metabolism pathways (Table 1) [85].

Vitamin D is a significant factor in early pregnancy, influencing maternal–fetal immune responses and genomic stability. It is associated with NK and B cell immunity, as well as the balance between Th1/Th2 cells. Insufficient vitamin D levels may increase autoantibody concentrations, including anti-phospholipid antibodies, significantly increasing miscarriage risk [86,87]. However, whether early diagnosis or vitamin D supplementation improves pregnancy outcomes in women with recurrent miscarriage remains uncertain [88]. Although a direct link between hyperhomocysteinemia and miscarriage has not been proven, some commonalities with other factors known to cause miscarriages, such as APS, have been observed [1,89]. Elevated homocysteine levels have also been suggested as a link between PCOS and RPL [90]. Consequently, homocysteine testing is not routinely recommended in clinical practice for women with RPL [1].

Metabolic differences, involving acylcarnitine, sphingolipid, and glycerophospholipid metabolism, in the decidual cells of women with RPL compared to non-pregnant women have been suggested, indicating that single or multiple metabolic pathway abnormalities play pivotal roles in RPL [41]. Low succinate accumulation and high expression levels of succinate dehydrogenase complex iron sulfur subunit B have been observed in chorionic villi from patients with RPL, inhibiting trophoblast invasion [42]. Metabolomic screening of blood samples using GC-MS has revealed increased lactic acid expression and decreased 5-methoxytryptamine expression in RPL [43]. The metabolomic profile of follicular fluid has been investigated in RPL, revealing elevated dehydroepiandrosterone levels, while other metabolites—25-hydroxyvitamin D3, phenylalanine, leucine, docosahexaenoic acid, and hydroxycholesterol—are decreased [44]. Despite these insights, limitations persist in metabolomic studies of RPL.

### 2.4. Prognostic Protein Biomarkers in RPL

Researchers have attempted to use proteomics tools to screen RPL-associated proteins across different tissue samples (Table 1) [17,46,48,49,51,52,71,91,92,93]. Proteomics, particularly the combination of 2-DE-LC-MS/MS and iTRAQ, is a useful method for detecting altered protein expression, as well as target proteins involved in disease pathogenesis, in comparison to conventional biochemical approaches that detect only one or a few specific proteins at a time. In a previous study, Kim et al. identified, for the first time, RPL-associated proteins in the follicular fluid of patients with RPL using 2-DE-LC-MS/MS-based proteomic tools [45]. This technique uses an electrical field to manipulate charged particles, separating protein mixtures in a fluid, which are subsequently examined spectroscopically. Among these proteins, coagulation factors such as antithrombin (AT) and fibrinogen-γ, crucial for maintaining normal pregnancy, have been validated. These markers improve our understanding of RPL pathogenesis and potentially enhance clinical management. Specifically, fibrinogen-γ, an essential component of the maternal coagulation system, is involved in early trophoblast cell proliferation and diffusion, contributing to the development of the feto–maternal microenvironment [94]. AT, a serine protease inhibitor, helps maintain blood fluidity. Women with AT deficiency face an increased risk of pregnancy-related venous thromboembolism, particularly when combined with other thrombophilic mutations, such as Factor V Leiden and prothrombin mutations [95]. However, evidence supporting the positive effects of anticoagulant treatment in women with hereditary thrombophilia remains inconclusive, necessitating large-scale international randomized controlled trials for effective treatment [1].

Moreover, a subsequent comparative proteomic study found a highly prevalent fragmented form of the protein inter-α-trypsin inhibitor heavy chain family member 4 (ITI-H4) in the serum of patients with RPL undergoing IVF treatment [46]. These results suggest that ITI-H4 expression could be used as a biomarker to detect RPL in patients by simply screening for the presence of proteins in the blood. The known cellular functions of ITI-H4 are critical in infectious responses [17]. Additionally, obese women with RPL have an altered endometrial protein profile, mainly related to increased haptoglobin expression, an inflammatory marker that might contribute to a higher risk of recurrent miscarriage [47].

Moreover, a recent large-scale proteomic analysis of placental villous samples identified angiotensinogen, mitogen-activated protein kinase 14 (MAPK14), and prothrombin (F2) as critical factors for early embryonic development [91]. Angiotensinogen, predicted to be the most significant upstream regulator, influences embryonic development through the renin–angiotensin system, while abnormalities in F2 are related to thrombosis, increasing the risk of RPL [92,96]. MAPK14 is involved in MAPK signaling pathways and mediates embryonic responses and apoptosis [97]. Integrated proteomic studies on decidual samples obtained from patients with normal early-stage pregnancies and those with RPL have revealed upregulated expression levels of NDUFB3 and COX-2 proteins, which are involved in apoptosis and oxidative stress [48]. Notably, proteins related to the regulation of endothelial cell proliferation and migration, as well as coagulation, including enolase 1 (ENO1) and calumenin, were found to be downregulated in villi from patients with unexplained recurrent miscarriage compared to normal controls [50]. However, conflicting findings have been reported, such as the abundance of ENO1 in the placenta of patients with unexplained recurrent miscarriages [98]. Despite advances in proteomics technology, very few RPL-related markers have been validated or used clinically.

## 3. Potential Biomarkers and Functional Pathways Related to the Pathogenesis of RPL

**Figure 3:**
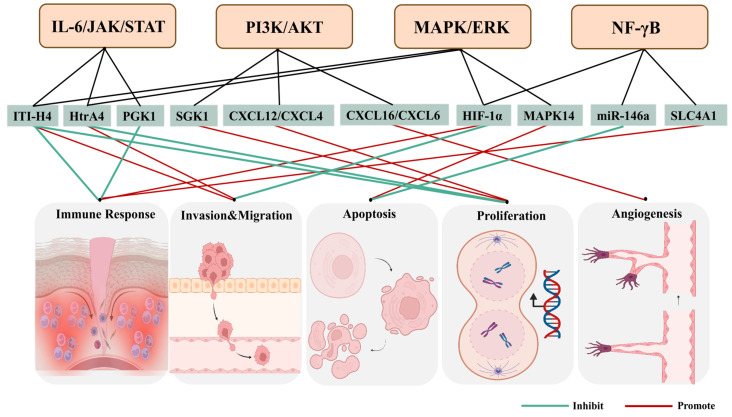
Different genes and gene products regulate cellular processes involved in the pathogenesis of RPL through diverse signaling pathways. Based on their characteristics and biochemical studies of related factors, it has been proposed that different signaling molecules are required for the coordination of several intercellular communications and maintenance of pregnancy, such as the PI3K/AKT and IL-6/STAT3 pathways. Moreover, the aberrant activation or repression of these signaling networks could lead to recurrent miscarriage events.

Omics studies have extensively investigated the numerous risk factors involved in RPL. However, the precise functions of the implicated genes and gene products in recurrent miscarriages are yet to be fully understood. To unravel the molecular intricacies of RPL, a signaling pathway analysis was employed to gain a comprehensive understanding of its pathogenesis. The abnormal activation or repression of multiple signaling pathways has been identified in cases of pregnancy loss, including the PI3K/AKT, MAPK/ERK, IL-6/STAT3, IL-1β/NF-κB, HIF-1α/VEGF, RhoA/ROCK2, and Shh/Gli pathways (Figure 3) [99,100,101,102,103]. Herein, we discussed the main insights gained into how the identified targets are components of specific pathways that may play a role in recurrent miscarriages. We explored the correlations and overlaps between these molecular pathways and discussed how these critical signaling pathways could contribute to miscarriage.

### 3.1. IL-6/JAK/STAT Pathway

Interactions between signal transducers and activators of transcription (STAT) and various upstream and target genes lead to numerous biological effects [104]. During early gestation, IL-6 activates the STAT signaling pathway by binding to receptors. Li et al. demonstrated that ITI-H4, identified as a biomarker of RPL, can be modulated by plasma kallikrein through the IL-6 signaling network, indicating a novel regulatory mechanism for inflammation in RPL [17]. Plasma kallikrein, a serine protease with diverse modulatory functions including thrombosis and inflammation, plays a crucial role in this process. IL-6 can increase plasma kallikrein levels and stimulate the production of ITI-H4 (∆N^688^) in a STAT/MAPK-dependent manner, leading to a decrease in the levels of ITI-H4 long isoform. Functional analysis showed opposing roles of the long isoform of ITI-H4 and ITI-H4 (ΔN^688^) in immune modulation, trophoblast cell invasion, immune cell migration, and cell proliferation [17]. Phosphoglycerate kinase 1 (PGK1) positively regulates ITI-H4 and inhibits the cleavage of the long isoform of ITI-H4 [105]. Consequently, the PGK1-ITIH4 axis may be related to the JAK2/STAT3 signaling pathway, offering potential insights into the pathogenesis of ITI-H4-related inflammatory diseases.

### 3.2. PI3K/AKT and MAPK/ERK Pathway

In addition, the PI3K-Akt and MAPK/ERK cascades play pivotal roles in mediating trophoblast survival, growth, and invasion in response to various extracellular signals during different phases of pregnancy [106]. Several studies have highlighted the involvement of the PI3K/Akt signaling pathway in the development of RPL [107,108,109]. The inhibition of PI3K/AKT promotes serum and glucocorticoid-regulated kinase 1 activity in endometrial cells during the receptivity window, disrupting embryo implantation in vivo [101]. Quantitative in vitro and in vivo experiments, involving the treatment of cultured endothelial progenitor cells with soluble Flt1, demonstrated a reduction in the frequency of miscarriage through the VEGF-PI3K/Akt-endothelial nitric oxide synthase pathway in a mouse recurrent miscarriage model [110]. Abnormal chemokine expression has been correlated with various pregnancy-related conditions, including preeclampsia, recurrent implantation failure, recurrent miscarriage, and fetal growth restriction. For instance, chemokine ligand 12 (CXCL12) has been implicated in promoting the invasion and migration of endometrial epithelial cells through CXCR4 ligation, activating the PI3K/AKT pathway in an autocrine manner at the maternal–fetal interface [111]. Moreover, the MAPK/ERK pathway has been associated with RPL. ERK1/2 inactivation results in decreased cell proliferation, affects embryo implantation during early pregnancy, and contributes to the maintenance of a normal pregnancy [112]. These findings underscore the intricate involvement of the PI3K/AKT and MAPK/ERK pathways in the pathogenesis of RPL and shed light on potential therapeutic targets and diagnostic markers for this complex reproductive disorder.

### 3.3. NF-κB Pathway

Moreover, key components of the NF-κB pathway have been identified in pregnancy tissues. Moreover, elevated progesterone, a hormone secreted during pregnancy, suppresses NF-κB activity [113]. Dysregulation of the NF-κB signaling pathway is a crucial factor in RPL pathogenesis and regulates Th1/Th2 immune responses [114]. Similarly, anti-inflammatory cytokines like IL-10, pivotal in the Th2 immune response, downregulate NF-κB at the maternal–fetal interface, potentially facilitating successful placentation in normal pregnancy [71]. Apoptosis is another crucial process for normal placental development and differentiation and maintaining a homeostatic environment [115]. Intriguingly, the transcription factor NF-κB exerts negative regulation in maternal peripheral blood T cells, playing a protective role against apoptosis [116]. Furthermore, several studies have indicated a crosstalk between the NF-κB and hypoxia-inducible factor (HIF) signaling pathways and their regulatory roles during pregnancy [117]. For example, HIF-1α, a major oxygen-sensing transcription factor, is directly regulated in an NF-κB-dependent manner, and the NF-κB-HIF-1α-MMP-2/9 axis regulates autophagy to modulate trophoblastic invasion [118].

### 3.4. Others

Other signaling mechanisms and oxidative stress have been implicated in the etiology of RPL, mediating adverse pregnancy outcomes through altered inflammation and angiogenesis [119,120]. Dysfunction in the Shh/Gli signaling pathway that activates autophagy reportedly inhibits trophoblast migration and angiogenesis, presenting another potential therapeutic target for RPL [121]. Additionally, results from the Kyoto Encyclopedia of Genes and Genomes network pathway analysis of RNA-Seq expression profiles of normal and RPL endometrial tissues have revealed significant alterations in nearly 1174 genes and associated biological pathways in the endometria of patients with RPL. Further studies have identified candidates that mediate overlapping intracellular signaling cascades, including ribosome oxidative phosphorylation [103]. Thus, it is conceivable that various markers comprise a hierarchical signaling network and that their related downstream signaling cascades converge to regulate gene or protein expression. Alternatively, these molecules might function in a signaling pathway initiated by cell surface receptors, leading to receptor recycling, which activates downstream targets that ultimately regulate gene transcription or directly or indirectly influence the relevant gene promoter function.

## 4. Pathways and Targeted Therapeutic Strategies for Recurrent Miscarriage

Adiponectin has been identified to regulate the Th17/Treg balance via the signal transducer and activator of the transcription 5 (STAT5) signaling pathway, thereby reducing the risk of miscarriage in animal models [122]. Anzi Heji (AZHJ), a traditional Chinese medicinal compound, was initially developed for the treatment of anti-cardiolipin antibodies-positive pregnant women to improve pregnancy hormone levels. Interestingly, the inhibition of DNA methyltransferases promoted trophoblast cell proliferation via the activation of the JAK/STAT pathway following treatment with AZHJ [123]. Additionally, granulocyte colony-stimulating factor (G-CSF) has been demonstrated to effectively reverse RPL-related traits by regulating the JAK1/STAT3 and mitogen-activated protein kinase (MAPK) signaling pathways in JEG-3 cells [124]. A baicalin regimen has been reported to reverse the differentiation of conventional dendritic cells through STAT5/ID2 signaling in decidual tissues [125]. Moreover, Cho-kyung-jong-ok-tang, a traditional Korean formula, could promote natural killer cell differentiation and prevent miscarriage by activating the STAT6/GATA3 pathway [126].

Icariin (ICA), a naturally prenylated flavonol glycoside extracted from plant epimedium, may alleviate osteoarthritis by reducing autophagy and inhibiting the PI3K/AKT/mTOR signaling pathway. Upon treatment with ICA, the expression of pro-inflammatory factors is reduced via the mTOR pathway, thereby improving the outcome of recurrent miscarriage [127]. Other formulations, such as Bushen Huoxue, exert beneficial effects by targeting the PI3K/AKT signaling pathway [128].

In addition to the treatment of autoimmune diseases such as systemic lupus erythematosus, hydroxychloroquine (HCQ) has many beneficial effects based on its vasculoprotective and antithrombotic properties. The use of HCQ has a good safety record and improves pregnancy losses via the EKR5 and NF-κB pathways [129]. Interestingly, immunosuppressive treatment with cyclosporin A improved pregnancy outcomes in JAR cells by activating MAPK/ERK signaling, thus upregulating the invasive potential of trophoblast cells [130].

Kidney-replenishing herbs are traditional medicinal formulas that have been widely used for the clinical treatment of RPL. A recent study suggests that kidney-replenishing herbs can upregulate suppressors of cytokine signaling via ERK/MAPK signaling, enhancing the growth of trophoblast cells [131]. Further work on kidney-replenishing herbs needs to be performed, which would be useful in the treatment of recurrent miscarriages.

Furthermore, interleukin IL-23 inhibits trophoblast proliferation and migration by activating the MAPK signaling pathway to promote miscarriage, which suggests that IL-23 might be a novel target for the treatment of RPL [132].

Overall, based on the available studies conducted in China and other Asian countries with different herb compositions, whether herbal treatments are equally effective and safe for Western populations remains questionable.

## 5. Potential Clinical Implications and Possible Applications

To address the heterogeneity of RPL definitions, there is a need for scientifically justified criteria [9]. Diagnosing and treating URPL poses particular challenges owing to the lack of expert consensus in this area. Utilizing a molecular marker could improve patient counseling and management, aiding in achieving a successful pregnancy and providing information about the risks of delivering a baby with health complications. Because the genetic causes of most RPL cases are likely to be private or rare in the general population, it is imperative to reconsider strategies to maximize the number of cases. Combining pedigree-based methods with novel NGS-based genome analysis methods is necessary to uncover rare and unique mutations in couples experiencing early pregnancy loss. This requires the establishment of dense collaboration among obstetricians, pediatricians, and geneticists to uncover the pathogenic mechanisms underlying this complex clinical phenotype within families. Currently, various transcriptomics-based tools are encouraged for use in clinical practice to incorporate personalized embryo transfer; however, none of the available diagnostic workups are applicable to RPL cases in clinics. Extensive proteomics research on early pregnancy loss has identified several key differentially expressed proteins associated with RPL. Translating these biomarkers into clinical settings has the potential to revolutionize the field of diagnostics and therapeutics. Ideally, this requires the establishment of collaborative multi-center efforts and the standardization of methodologies with substantial resources.

Recent developments in placental organoid technology, patient-specific endometrial models, and the high-throughput single-cell sequencing of tissue samples can be utilized for multi-omics characterization [133,134,135]. This advancement is expected to enhance our understanding of the sequence of events that leads to RPL and advance the development of new clinical therapeutics.

## 6. Conclusions

RPL is a multifaceted condition wherein many clinical cases remain enigmatic despite known chromosomal and immunological abnormalities. Combining findings from multi-omics approaches and functional characterization is essential for unraveling the underlying mechanisms. More stringent and standardized clinical definitions, along with increased cooperation among experts, are required to effectively manage couples experiencing RPL.

Recent advancements in omics studies have identified various factors associated with RPL (Table 1), revealing intricate underlying biological mechanisms. Understanding the various functions of gene transcripts has led to advancements in biomarker identification and drug development (Table 2). In this review, we have explored recent insights into the biological functions of these markers gleaned through genetic and biochemical studies. However, several questions remain unanswered. The roles of epigenetic and fetal metabolic programming in pregnancy success and early loss are unclear. Additionally, it is uncertain whether target genes or proteins are fundamental molecules in RPL progression. RPL development involves several complex mechanisms regulated by multiple molecules and pathways.

While the utilization of state-of-the- art “omics” analysis tools to analyze biomaterials from recurrent miscarriage cases is currently in the planning stage, these findings hold promise for uncovering novel clinical biomarkers and elucidating the cellular and molecular mechanisms underlying RPL. Novel treatment options targeting critical biomarkers may emerge from these endeavors, addressing the need for a deeper understanding of the mechanisms underlying RPL.

Determining if a target marker affects the expression of other genes in targeted therapy requires additional clinical and experimental evidence. A systematic screening approach and detailed exploration of gene, protein, and metabolite interactions are essential for a comprehensive understanding of RPL causes. Further multi-omics-based research, coupled with rigorous functional studies, will significantly contribute to identifying drug targets and discovering biomarkers, ultimately enhancing our ability to address and manage RPL.

## Figures and Tables

**Table 1:** Molecules associated with omics-based RPL studies.

Omics	Biomarker	Samples	Functionality	Sample Population	References
Genomics (Metagenomics)	FGF9, FGF9, TLE1, TLE4, E2F8, SIK1	Endometrial tissue	Angiogenesis, trophoblast differentiation	Europe, China, Africa, USA	[18]
Genomics (NGS)	DYNC2H1, KIF14, RYR1, GLE1	Serum/plasma	Cell division, cilia function, or fetal movement	China, Switzerland, UK	[21]
Genomics (WES)	TLE6, NLRP7, FSHR, ZP1, NLRP5, CBX3, PIF1, PLK1	Serum/plasma	Early embryonic development, oocyte maturation, and DNA replication	Saudi Arabia	[22]
Genomics (GWAS)	MHC	Serum/plasma	Immune response	Japanese	[23]
Genomics (WES)	PIEZO1, FRAS1, COL1A, POMT1, DIS3L2, BBS12	Serum/plasma	Cell division, proliferation, differentiation, apoptosis, and migration	Iran	[24]
Genomics (NGS)	ALPP, FOXD1, HLA-G, C4BPA	Serum/plasma	Cell proliferation, division, and immune response	USA, India, China, Brazil	[25]
Epigenetics (Methylation)	LINE-1	Serum/plasma	Cell proliferation, division, and immune response	Italy	[16]
Epigenetics (Methylation)	DNMTs, TETs	Chorionic villi	Immune tolerance, cell invasion	China	[26,27]
Epigenetics (Methylation)	H3-K9, G9aMT	Decidual/ endometrial tissue	Embryonic development	India	[28]
Epigenetics (Methylation)	GRB10, IGF2	Placental tissue	Fetal metabolic, neurological, and embryo development	Russia	[29,30]
Epigenetics (Methylation)	CREB5, RBM24, IRF4, DPYSL4	Decidual tissue	Cell migration, apoptosis	China	[31]
Epigenetics (miRNAs)	miR-25, miR-32, miR-125, and miR-222	Serum/plasma	Angiogenesis, vasculogenesis, and apoptosis	Korea	[32,33]
Epigenetics (IncRNAs)	Lnc-SLC4A1-1	Chorionic villi	Immune response, cell migration, and apoptosis	China	[34]
Epigenetics (IncRNAs)	TCL6, Lnc-49a, Lnc-HZ08, Lnc-HZ01	Chorionic villi	Cell proliferation, migration, and apoptosis	China	[35]
Transcriptomics (SSH)	hCG, PP14, MUC1, HtrA4	Chorionic villi	Immune response, trophoblast invasion, migration, and fusion	Korea	[14,36]
Transcriptomics (microarray)	TRAIL, S100A8	Decidual/ endometrial tissue	Immune response	UK	[37,38]
Transcriptomics (RNA-Seq)	E2F/DP1	Chorionic villi	Placental development, fetal viability	Estonia	[39]
Transcriptomics (scRNA-seq)	TNFSF12, FASLG	Decidual tissue	Macrophages and NK cells activation	China	[40]
Metabolomics	Acylcarnitine, sphingolipid	Decidual tissue	Trophoblast differentiation, placental angiogenesis	China	[41]
Metabolomics	Succinate, SDHB	Chorionic villi	Trophoblast invasion and proliferation	China	[42]
Metabolomics	Lactic acid, 5methoxytryptamine	Plasma	Inflammation	China	[43]
Metabolomics	Dehydroepiandrosterone, 25-hydroxyvitamin D3, phenylalanine, leucine, docosahexaenoic acid, and hydroxycholesterol	Follicular fluid	Inflammation	China	[44]
Proteomics	AT, fibrinogen-γ	Follicular fluid	Thrombosis, fetal growth	Korea	[45]
Proteomics	ITI-H4, KLKB1	Serum	Immune tolerance, trophoblast invasion, proliferation, and migration	Korea	[17,46]
Proteomics	Haptoglobin	Endometrial tissue	Inflammation	UK	[47]
Proteomics	NDUFB3	Decidual tissue	Oxidative stress	China	[48]
Proteomics	DICER1, CDK2, PBX1, MAPK14, Lamin B1	Decidual tissue	Self-renewal of human embryonic stem cells	China	[49]
Proteomics	Calumenin, prohibitin, apolipoprotein A-I	Placental tissue	Cell proliferation, angiogenesis, and coagulation	Iran	[50]
Proteomics	CD45, PSG1, Prdx-2	Serum	Oxidative damage	China	[51]
Proteomics	IGFBP-rp1/IGFBP-7, Dkk3, RAGE and angiopoietin-2	Serum	Apoptosis, inflammation, angiogenesis, invasion, and vascular remodeling	China	[52]
Proteomics	MMP10, CEMIP, ANK3	Chorionic villi	Fetal development	Estonia	[39,49]

Abbreviations: FGF9, fibroblast growth factor 9; TLE, transducer-like enhancer of split; E2F8, E2F transcription factor 8; SIK1, salt-inducible kinase; DYNC2H1, dynein cytoplasmic 2 heavy chain 1; KIF14, kinesin family member 14; RYR1, ryanodine receptor 1; GLE1, gametic lytic enzyme 1; NLRP, nucleotide-binding oligomerization domain, leucine rich repeat and pyrin domain containing; FSHR, follicular-stimulating hormone receptor; ZP1, zona pellucida glycoprotein 1; CBX3, chromobox 3; PLK1, polo like kinase 1; PIEZO1, piezo type mechanosensitive ion channel component 1; FRAS1, fraser syndrome 1; COL1A, collagen type 1 α 1 chain; POMT1, protein o-mannosyl-transferase 1; DIS3L2, DIS3-like exonuclease 2; BBS12, bardet-biedl syndrome 1; ALPP, alkaline phosphatase, placental; FOXD1, forkhead box 1; HLA-G, human leukocyte antigen G; C4BPA, complement component 4 binding protein α; DNMTs, DNA methyltransferases; TETs, ten-eleven translocation enzymes; H3-K9, methylated histone; G9aMT, methyl transferase; GRB10, growth factor receptor bound protein 10; IGF2, insulin-like growth factor 2; CREB5, cAMP responsive element binding protein 5; RBM24, RNA-binding protein 24; IRF4, interferon regulatory factor 4; DPYSL4, dihydropyrimidinase like 4; TCL6, T-cell leukemia/lymphoma 6; hCG, human gonadotrophin; PP14, placental protein 14; MUC1, mucin 1; HtrA4, HtrA serine peptidase 4; TRAIL, TNF-related apoptosis-inducing ligand; S100A8, S100 calcium-binding protein A8; TNFSF12, TNF superfamily member 12; FASLG, fas ligand; SDHB, succinate dehydrogenase complex iron sulfur subunit B; AT, anti-thrombin; ITI-H4, ITI-H4, inter-α-trypsin inhibitor heavy chain family member 4; KLKB1, plasma kallikrein; NDUFB3, NADH dehydrogenase (ubiquinone) 1 β subcomplex, 3; DICER1, dicer 1, ribonuclease III; CDK2, cyclin dependent kinase 2; PBX1, pre-B-cell leukemia transcription factor 1; MAPK14, mitogen-activated protein kinase 14; PSG1, Präzisionsschützengewehr-1; Prdx-2, peroxiredoxin-2; IGFBP, insulin-like growth factor-binding protein; Dkk3, dickkopf-related protein 3; MMP, matrix metalloproteinases; CEMIP, cell migration-inducing and hyaluronan-binding protein; ANK3, ankyrin-3.

**Table 2:** The mechanism for drug treatment of RPL targeting different pathways.

Drug	Species, Tissues, or Cells	Pathways	Mechanism	References
Recombinant adiponectin	Mouse decidua	STAT5	Regulate STAT5 signaling pathway to maintain Th17/Treg balance	[122]
AZHJ	Mouse decidua; HTR-8/SVneo cells	JAK/STAT	Promote trophoblast cell proliferation via JAK/STAT pathway	[123]
G-CSF	JEG-3 cells	JAK1/STAT3	Activate JAK/STAT and MAPK pathways in trophoblast cells	[124]
Baicalin	Mouse decidua	STAT5	Inhibit STAT5/ID2 pathway in dendritic cells of decidua, suppressing their differentiation	[125]
CKJOT	Mouse decidua	STAT6	Activate STAT6/GATA3 signaling pathway to promote transformation of NK2 cells	[126]
ICA	Mouse decidua	PI3K/AKT/mTOR	Inhibit mTOR pathway	[127]
BSHXD	Mouse decidua	PI3K/AKT	Immune response	[128]
HCQ	Clinical studies	EKR5 and NF-γB pathways	Inhibit NF-κB pathways	[129]
Cyclosporin A	JAR cells	MAPK/ERK	Activate MAPK/ERK pathways in JAR cells	[130]
IL-23	HTR-8/SVneo cells	P38/MAPK	Inhibit trophoblast proliferation and migration via MAPK pathway	[132]

Abbreviations: AZHJ, Anzi Heji; G-CSF, granulocyte colony-stimulating factor; CKJOT, Cho-kyung-jong-ok-tang; ICA, Icariin; BSHXD, BuShen HuoXue Fang; HCQ, Hydroxychloroquine; IL-23, interleukin-23.

## Data Availability

All data supporting the findings of this study are available within the paper.

## Abbreviations

The following abbreviations are used in this manuscript.

RPL	Recurrent pregnancy loss
GWAS	Genome-wide association study
DNMTs	DNA methyltransferases
TETs	Ten-eleven translocation enzymes
MTHFR	Methykenetetrahydrofolate reductase
hCG	Human gonadotrophin
miRNAs	microRNAs
HtrA4	High-temperature requirement factor A4
PCOS	Polycystic ovarian syndrome
AT	Antithrombin
ITI-H4	Inter-α-trypsin inhibitor heavy chain family member 4
MAPK14	Mitogen-activated protein kinase 14
ENO1	Enolase 1
STATs	Signal transducers and activators of transcription
PGK1	Phosphoglycerate kinase 1
